# Widespread changes in nucleosome accessibility without changes in nucleosome occupancy during a rapid transcriptional induction

**DOI:** 10.1101/gad.293118.116

**Published:** 2017-03-01

**Authors:** Britta Mueller, Jakub Mieczkowski, Sharmistha Kundu, Peggy Wang, Ruslan Sadreyev, Michael Y. Tolstorukov, Robert E. Kingston

**Affiliations:** 1 Department of Molecular Biology, Massachusetts General Hospital, Harvard Medical School, Boston, Massachusetts 02114, USA;; 2 Department of Pathology, Massachusetts General Hospital, Harvard Medical School, Boston, Massachusetts 02114, USA

**Keywords:** chromatin, MNase, nucleosome, occupancy, UPR, enhancers

## Abstract

Activation of transcription requires alteration of chromatin by complexes that increase the accessibility of nucleosomal DNA. Removing nucleosomes from regulatory sequences has been proposed to play a significant role in activation. We tested whether changes in nucleosome occupancy occurred on the set of genes that is activated by the unfolded protein response (UPR). We observed no decrease in occupancy on most promoters, gene bodies, and enhancers. Instead, there was an increase in the accessibility of nucleosomes, as measured by micrococcal nuclease (MNase) digestion and ATAC-seq (assay for transposase-accessible chromatin [ATAC] using sequencing), that did not result from removal of the nucleosome. Thus, changes in nucleosome accessibility predominate over changes in nucleosome occupancy during rapid transcriptional induction during the UPR.

## Abstract (precis)

In this study, Mueller et al. investigated whether changes in nucleosome occupancy occurred in the set of genes that is activated by the unfolded protein response (UPR). Their findings demonstrate no decrease in occupancy on most promoters, gene bodies, and enhancers, but instead there was an increase in the accessibility of nucleosomes, as measured by MNase digestion and ATAC-seq, that did not result from removal of the nucleosome.

Many sequence-specific DNA-binding regulatory factors and components of the general transcription machinery are inhibited by nucleosome formation (Kornberg 1974). During transcriptional activation, nucleosomes have been proposed to either slide along the DNA to free regulatory sites or be removed by transfer of the histones to a chaperone or another region of DNA (Peterson and Workman 2000; Narlikar et al. 2002; Clapier and Cairns 2009). The identification of putative nucleosome-free regions at transcription start sites (TSSs), in enhancer sequences, and in regulatory regions has led to proposals that removal of nucleosomes from regulatory sites might be a key mechanism in activation (Yuan et al. 2005; Mavrich et al. 2008). Alternative mechanisms include altering the accessibility of DNA on the surface of the nucleosome by ATP-dependent remodelers, replacement with certain histone variants, or destabilization of internucleosome contacts by acetylation of lysine residues. These latter models have received support from recent studies identifying “fragile” nucleosomes near certain TSSs, transiently destabilized nucleosomes during viral induction of gene expression, and retention of nucleosomes in enhancer regions bound by FoxA and other factors (Xi et al. 2011; Knight et al. 2014; Sexton et al. 2014; Teif et al. 2014; Soufi et al. 2015; Iwafuchi-Doi et al. 2016). A genome-wide analysis concerning which of these classes of mechanisms might predominate during acute regulation in complex eukaryotes has not been performed due to the inherent difficulties in measuring nucleosome occupancy and accessibility across large genomes.

We measured nucleosome occupancy and nucleosome accessibility genome-wide in *Drosophila* cells during the unfolded protein response (UPR), an evolutionarily conserved response to protein misfolding that changes the regulation of hundreds of genes (Hollien and Weissman 2006). We used the UPR as a model because it involves widespread changes and is important to numerous aspects of physiology. The UPR is a stress response pathway that senses changes in endoplasmic reticulum (ER) protein folding, calcium homeostasis, or ER membrane integrity. The three ER stress sensors IRE1, PERK, and ATF6 are located in the ER membrane. When triggered, they activate three transcription factors (TFs): XBP-1, ATF4, and a cleaved non-membrane-bound fragment of ATF6. IRE1 splices the mRNA of XBP-1 to generate the active TF XBP-1s. The kinase PERK phosphorylates eIF2α (a translation initiation factor) to stall general translation but promote translation of the TF ATF4. ATF6 travels to the Golgi, where it is cleaved, releasing the cytosolic version of ATF6, which harbors the TF domain. The three TFs translocate to the nucleus to initiate transcription of UPR target genes as part of an integrated coregulated program. If ER homeostasis cannot be re-established, the UPR will induce apoptosis (Walter and Ron 2011). Several human diseases have been implicated in aberrant UPR activation; e.g., cancer cells activate the UPR to survive stress conditions that occur inside the tumor microenvironment, such as hypoxia and poor nutrient availability (Grootjans et al. 2016). Understanding the mechanisms that regulate the UPR might allow modulation of this response.

We used a method involving titration of micrococcal nuclease (MNase) to map nucleosomes during a short (4-h) time course of activation of the UPR. The use of several MNase concentrations allowed us to capture nucleosomes that are preferentially released at both high and low levels of MNase, providing a more comprehensive occupancy map than is likely to be obtained at a single MNase amount (Iwafuchi-Doi et al. 2016; Mieczkowski et al. 2016; Chereji et al. 2017). These experiments, further supported by experiments performed using ATAC-seq (assay for transposase-accessible chromatin [ATAC] using sequencing), allowed us to determine whether a nucleosome was accessible or inaccessible in addition to its genomic location. We found that, in promoters and enhancers, predominant changes during the UPR occurred in nucleosome accessibility, not in nucleosome occupancy.

## Results

**Figure 1.**
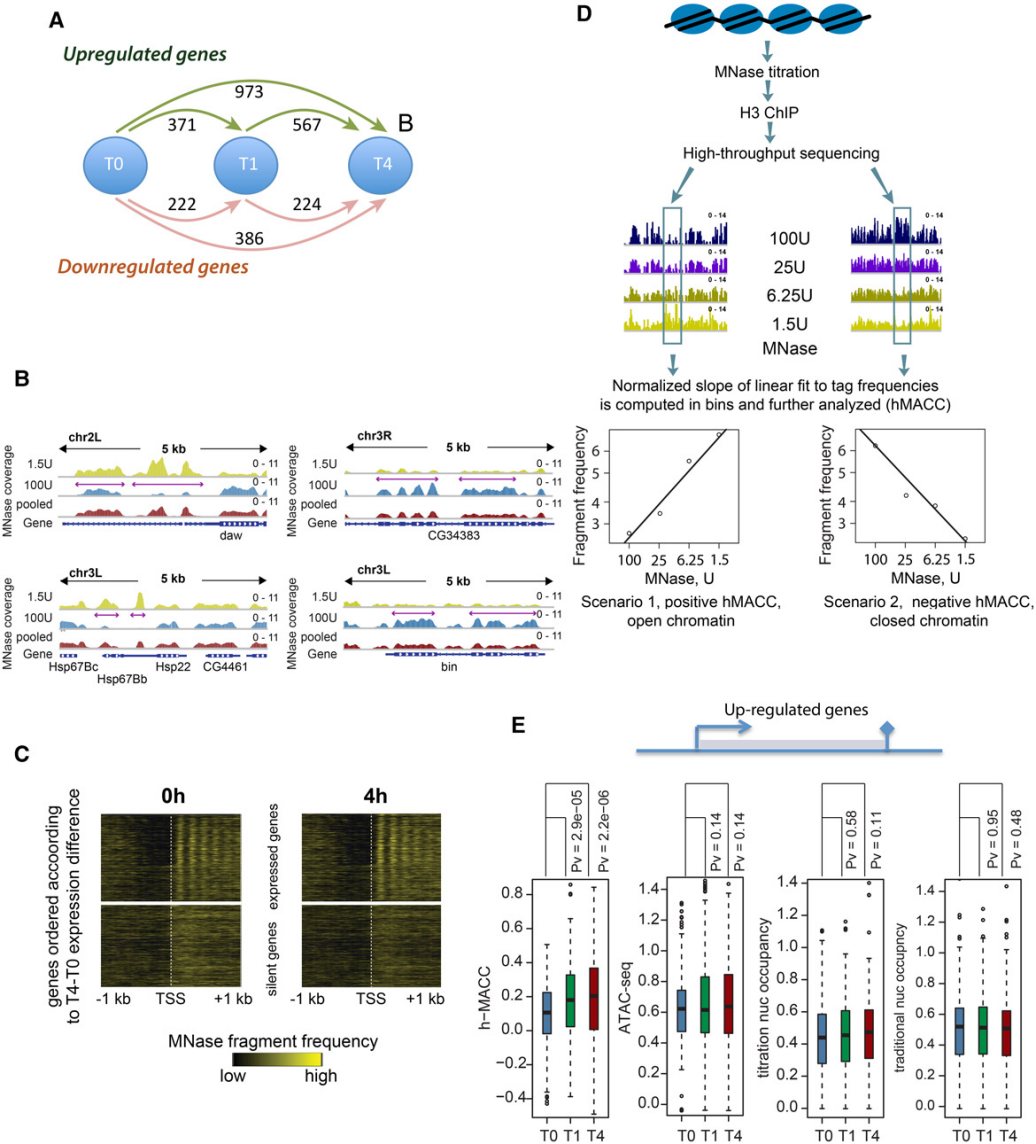
The UPR as a model for changes in chromatin states. (*A*) Schematic illustration of number of up-regulated (green arrows) and down-regulated (pink arrows) genes. (*B*) Examples of variable nucleosome positioning depending on MNase concentration. The *left* panels show examples of locations where nucleosome occupancy profiled with high MNase concentration (100 U) was lower than the occupancy profiled with lower MNase concentration (1.5 U). The *right* panels show the opposite situation. (*C*) Nucleosomal phasing into the gene bodies of active and silent genes. (*D*) The work flow of the MNase accessibility (MACC) assay. Chromatin fragments released by four MNase digestions of increasing depth were enriched by H3 chromatin immunoprecipitation and subjected to massively parallel sequencing. The normalized frequencies of the sequenced reads were assessed genome-wide in 200-base-pair (bp) bins. The slope of the regression line fitted on these frequencies was corrected for GC content bias and used as a measure of chromatin accessibility. (*E*) Per-gene levels of the MNase chromatin accessibility, ATAC-seq, titration occupancy and “traditional” occupancy over gene bodies computed for the up-regulated genes. (Blue box plot) Results for the 0-h time point; (green box plot) results for the 1-h time point; (red box plot) results for the 4-h time point. The significance of the changes was evaluated with the Mann-Whitney test.

We induced the UPR in *Drosophila* S2 cells with 5 mM dithiothreitol (DTT), a reducing agent that prevents disulfide-linkages in proteins, thereby inducing misfolding. To identify time points at which we expected nucleosomes to change during the UPR, we looked at UPR initiation in S2 cells by measuring splicing of the key UPR TF XPB-1 (Supplemental Fig. S1A, B) and using RNA sequencing (RNA-seq) to examine genome-wide changes in regulation (Fig. 1A; Supplemental Fig. S1C). We chose 1 h and 4 h to capture the UPR process at an early time point (1 h) after spliced XBP-1 had been fully generated and at a later time point after spliced XBP-1 had been present for a few hours (Supplemental Fig. S1B). We observed hundreds of genes that were both significantly up-regulated and down-regulated at least twofold during that time course, indicating that treatment with DTT induces a rapid and general transcriptional response (Fig. 1A; Supplemental Fig. S1C).

### MNase titration as a tool to determine occupancy and accessibility

To compare nucleosome occupancy and accessibility, we used MNase digestion followed by high-throughput sequencing (MNase-seq). MNase cleaves the linker DNA between nucleosomes, and thus MNase-seq allows an estimate of the portion of the genome that is occupied by nucleosomes (Tolstorukov et al. 2010; Zhang and Pugh 2011). While using standard MNase-seq protocols, which have been applied effectively in organisms such as *Saccharomyces cerevisiae*, we noted variations in measuring nucleosome occupancy from experiment to experiment that confounded interpretation. We attributed this to the fact that nucleosomes are variably released by different MNase concentrations, changes that are likely to be influenced by H1 association, histone variants, and compaction (Iwafuchi-Doi et al. 2016; Mieczkowski et al. 2016; Chereji et al. 2017). We therefore measured nucleosome occupancy on genes that were regulated during the UPR over a range of MNase concentrations. We included a histone immunoprecipitation step in the protocol, as nucleosome-sized protection might result from nonhistone proteins as opposed to nucleosomes (Mieczkowski et al. 2016; Chereji et al. 2017). We found numerous examples of locations where nucleosome occupancy was low or undetectable when high MNase concentration (100 U) was used to release nucleosomes, yet occupancy was high at low MNase concentration (1.5 U) (examples in Fig. 1B, left panels). We also observed the opposite behavior in some locations (Fig. 1B, right panels). We noted previously that these same phenomena are seen in genomes during normal cell growth (Mieczkowski et al. 2016). We concluded that generating an occupancy map of nucleosomes during the UPR required measuring occupancy at a variety of MNase concentrations and integrating the data in order to appropriately capture nucleosomes that were released at different levels of MNase digestion (“pooled” tracks) (Fig. 1B).

We used a series of four increasing MNase concentrations (MNase titration) to profile nucleosomes, sequenced each individual titration point, and then combined (averaged) the data. We computed nucleosome occupancy throughout the genome before and during induction of the UPR. This measurement recapitulated known parameters for nucleosome occupancy, including decreased occupancy at TSSs and phasing of nucleosomes into the gene body (Fig. 1C; Supplemental Fig. 1D). We noted that, as we observed previously (Mieczkowski et al. 2016), comparison of the quantity of nucleosomes released at each individual MNase concentration in the titration series provided information on whether release of a given nucleosome required low levels of MNase (i.e., the nucleosome was accessible to MNase) or high levels (the nucleosome was comparatively inaccessible to MNase). For example, the observed occupancy of nucleosomes going into active gene bodies was higher when low MNase was used and lower when high MNase was used, indicating that nucleosomes in active gene bodies were more exposed and thus susceptible to MNase digestion, perhaps due to transcription of these regions (Supplemental Fig. S1D, left panels).

We previously described and extended here the use of this differential ability of nucleosomes to be released by MNase to quantify nucleosome accessibility (Mieczkowski et al. 2016). We found previously that increased accessibility to MNase occurs on active genes and therefore were interested in determining how that accessibility changed during an acute response on both active genes and the surrounding regulatory regions. For example, others have shown previously, in disparate organisms, that nucleosomes at gene starts and ends or enhancers can have increased accessibility (Kubik et al. 2015; Chereji et al. 2016, 2017; Iwafuchi-Doi et al. 2016). We wished to assess these features during a genome-wide induction. We quantified accessibility to MNase with a metric called MNase accessibility (MACC) (Fig. 1D; Mieczkowski et al. 2016). MACC uses the amount of nucleosome “reads” released at each interrogated genomic location at each MNase concentration to generate a slope across the titration points; the direction and intensity of the slope is used to calculate a metric that defines regions as having high nucleosome accessibility (positive MACC values) or low accessibility (negative MACC values) (see Fig. 1D). We combined MNase titration with histone H3 immunoprecipitation (h-MACC) to ensure that sequenced DNA was bound by histones as opposed to other regulatory factors. We measured h-MACC scores and hence the MACC of nucleosomes at every portion of the genome and used the same data set to determine the occupancy of nucleosomes at all sites (see below for examples).

### Changes in occupancy and accessibility genome-wide

**Figure 2.**
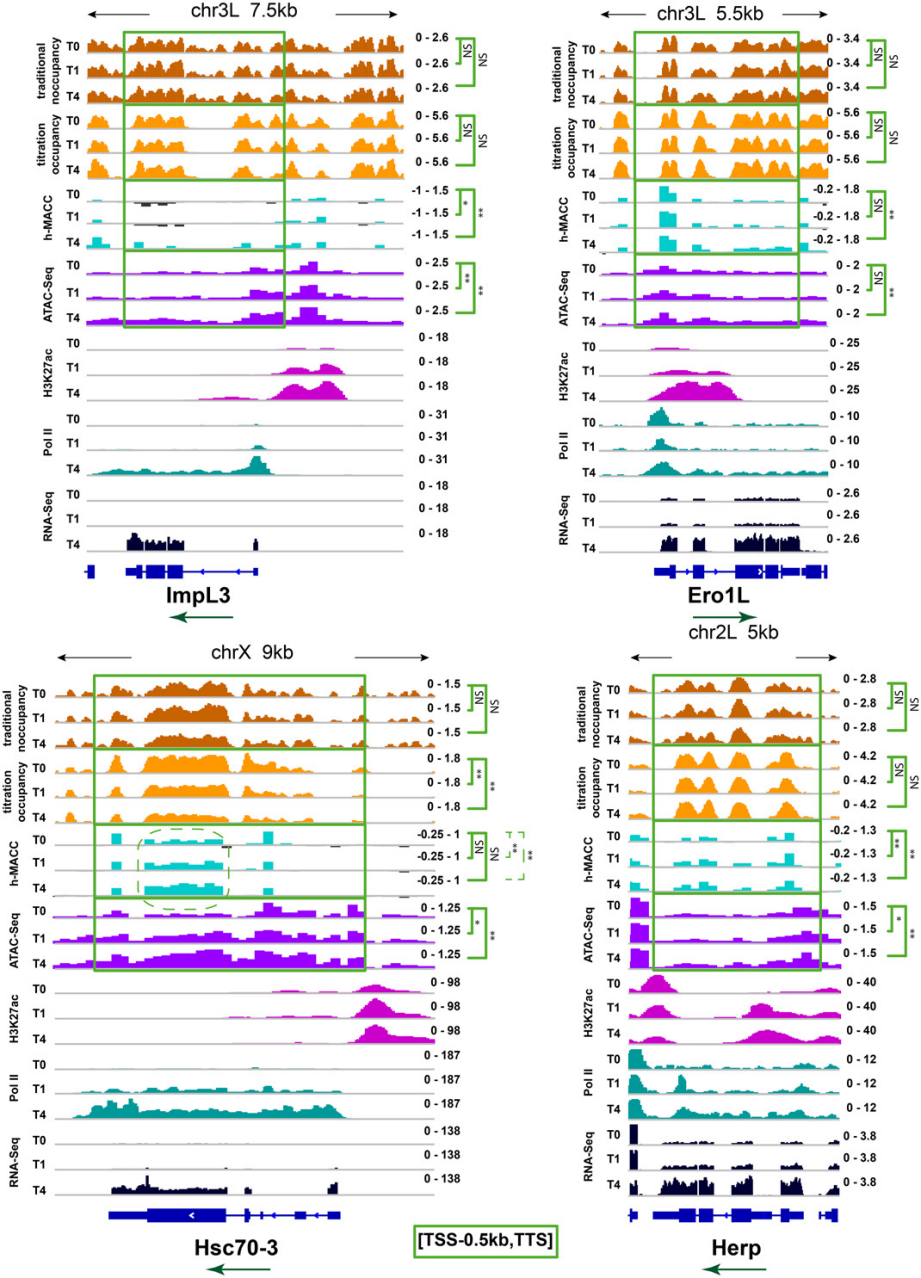
Examples illustrating chromatin structure arrangement and changes at up-regulated genes. Profiles of nucleosome occupancy, h-MACC, ATAC-seq, H3K27 acetylation (H3K27ac) enrichment, RNA polymerase II (Pol II), and RNA-seq at the loci encompassing selected up-regulated genes. The *top* panels show the profiles around genes *ImpL3* and *Ero1L*, and the *bottom* panels show the profiles around genes *Hsc70-3* and *Herp*. Metrics and time points are indicated at the *left* side of the plots. Directions of transcription are marked with green arrows. Similarly to Figure 1, the h-MACC and ATAC-seq profiles represent binned data. To show that binning did not remove important positional information from the occupancy data, the occupancy panels show nonbinned profiles (coverage). The significance of the difference between the 0-h (T0) and 1-h/4-h (T1/T4) profiles is shown for all four metrics at the *right* side of the plots (one-sided signed rank test). For both occupancy profiles, the significance of decrease was estimated, and for both accessibility profiles, the significance of increase was estimated; otherwise, tests were done in a similar manner for all of the profiles. The compared loci [TSS-500 bp, TTS] are highlighted with green boxes. (*) *P*-value < 0.05; (**) *P*-value < 0.01; (NS) not significant.

To determine whether nucleosomes changed position or were lost during the UPR, we focused on the genes that were at least twofold up-regulated between 0 and 1 h and either were further up-regulated between 1 and 4 h or did not further change their level of expression after 1 h. Three-hundred-twenty-seven genes fit these criteria. We were surprised to find that averaged occupancy did not change over these genes, as measured by MNase titration (Fig. 1E, middle panel, titration nucleosome occupancy). For the purposes of this analysis, we define “genes” as the region between the TSSs and transcription termination sites (TTSs); we analyze promoters and enhancers separately below. We validated this observation using standard MNase-seq protocols (Fig. 1E, right panel, traditional nucleosome occupancy). As an additional control, we used input-corrected measures of titration nucleosome occupancy and obtained similar results (Supplemental Fig. S1E). We show individual examples of genes and surrounding sequences in the following figures, demonstrating that, while there are some regions where occupancy changes, occupancy is surprisingly constant when examined either in total or by individual genes (Figs. 1E, 2).

In contrast, we found that accessibility on these up-regulated genes, as measured by MACC, increased significantly (Fig. 1E, left panel, h-MACC). We show individual examples of increasing accessibility in Figure 2 on genes that are up-regulated during the UPR. Surprisingly , occupancy does not change in these regions. As a control, we also examined bodies of the genes that do not change expression in the UPR (Supplemental Fig. S1F). As expected, chromatin accessibility and nucleosome occupancy did not change significantly in these genes between the UPR time points.

We compared the accessibility metric h-MACC with the accessibility metric generated by ATAC-seq (see the figures below). ATAC-seq uses a transposase, Tn5, to add sequencing adapters into native chromatin in vitro. Only the “open” chromatin structure will allow the transposase to deposit the sequencing adapters, whose location is determined by high-throughput sequencing (Buenrostro et al. 2013). We observed similar changes in accessibility with both h-MACC and ATAC-seq when analyzing individual loci (Fig. 2; Supplemental Fig. S1G). When analyzing total up-regulated genes, unlike h-MACC, ATAC-seq does not show a clear increase in gene bodies during the UPR (Fig. 1E, cf. the h-MACC and ATAC-seq panels). One possibility for this observation is that MACC might be more suitable for the detection of broad regions of change. We conclude that, in general, there are more changes in nucleosome accessibility than in nucleosome occupancy during the UPR.

We show examples of these phenomena on four individual genes where transcription increases during the UPR: *ImpL3*, *ERO1L*, *HSC70-3/BiP*, and *Herp* (Fig. 2). In each case, we compared occupancy as measured by titration and traditional protocols, accessibility as measured by h-MACC and ATAC-seq, the enhancer modification H3K27 acetylation (H3K27ac), RNA polymerase II (Pol II), and transcript levels. We note that chromatin accessibility was measured in 200-base-pair (bp) bins (as required by the MACC protocol), while occupancy was determined without binning the data (see the Materials and Methods). We confirmed that this difference did not affect our results, and the same conclusions can be reached for binned nucleosome occupancy (Supplemental Fig. S2A). We saw changes predominately in accessibility as measured by either h-MACC or ATAC-seq and some limited changes in occupancy (Fig. 2).

Highly transcribed genes, such as *HSC70-3/BiP* after induction, are expected to have fewer nucleosomes, as elongation by RNA Pol II is thought to disrupt the nucleosomal organization. We observed decreased nucleosome occupancy on the BiP gene during induction, concordant with this expectation (Fig. 2, *HSC70-3/BiP* panel, bottom left). These data demonstrate that the occupancy measurements that we used are able to detect decreases during the UPR. We did not see significant decreases in occupancy in either the other highly regulated genes examined in Figure 2 or the strongly up-regulated genes as a group (Supplemental Fig. S2B), indicating that the occupancy decrease observed with BiP is the exception as opposed to the rule during the UPR (see also Fig. 1E). For all four genes (Fig. 2), we observed widespread changes in accessibility as measured by both h-MACC and ATAC-seq in gene bodies and regulatory regions (see below). We conclude that changes in nucleosome accessibility predominated over changes in occupancy on regulated genes during the UPR.

### Promoter and enhancer regions display changes in accessibility

**Figure 3.**
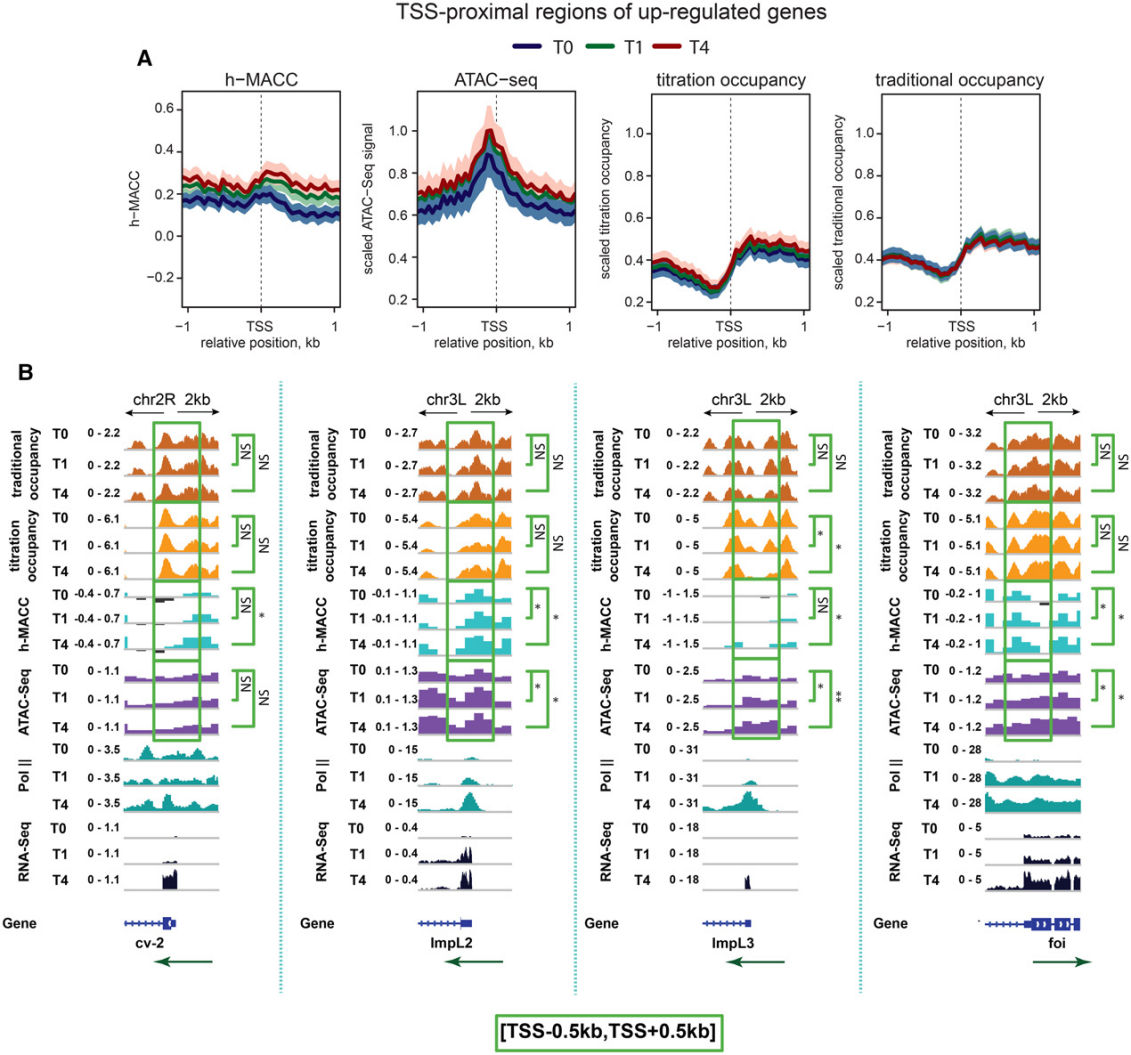
Chromatin organization at promoter regions of the genes up-regulated during the UPR. (*A*) Average profiles of chromatin accessibility and nucleosome occupancy characteristics around the TSSs of up-regulated genes. From *left* to *right*, results are shown for h-MACC, ATAC-seq, titration occupancy, and “traditional” occupancy. The thick lines correspond to the average profiles, while colored areas give reference of the standard errors of mean (SEM). (Blue) The 0-h time point; (green) the 1-h time point; (red) the 4-h time point. (*B*) Examples illustrating chromatin structure at individual promoters of up-regulated genes. Profiles of nucleosome occupancy, h-MACC, ATAC-seq, H3K27ac, Pol II, and RNA-seq are shown in each plot. The metrics are identified at the *left* of each plot, and the directions of transcription are marked with green arrows. The significance of the difference between T0 and T1/T4 profiles around the TSS (±500 bp) is indicated at the *right* (one-sided signed rank test) (see the legend for Fig. 2 for details).

It is plausible that occupancy changes are found primarily in promoter regions of up-regulated genes. To test this, we focused on TSS-proximal regions (promoters) of up-regulated genes, which we defined as regions within 1 kb of TSSs. There was no general decrease in occupancy on promoters of up-regulated genes (Fig. 3A, right two panels; Supplemental Fig. S3A, right two panels). In contrast, h-MACC and ATAC-seq scores increased across these regions (Fig. 3A, left two panels; Supplemental Fig. S3A, left panel). Consistent with these observations, h-MACC showed positive correlation with gene expression levels at all three time points, while occupancy showed a negative correlation (Supplemental Fig. S3B). In contrast, examination of promoters of genes whose expression was down-regulated during the UPR showed no significant changes in accessibility or occupancy (Supplemental Fig. S3C).

We examined individual promoters to look for evidence of a loss of nucleosomes at promoter regions. Four representative promoters all showed increased accessibility (Fig. 3B). Only one of these four promoters showed decreased occupancy (*ImpL3*) (Fig. 3B). In addition, there were no significant changes in nucleosome position detectable when analyzing the TSS-proximal regions of more up-regulated genes, four of which are shown here (Supplemental Fig. S3D). We conclude that increases in accessibility of nucleosomes are seen more generally than changes in occupancy.

**Figure 4.**
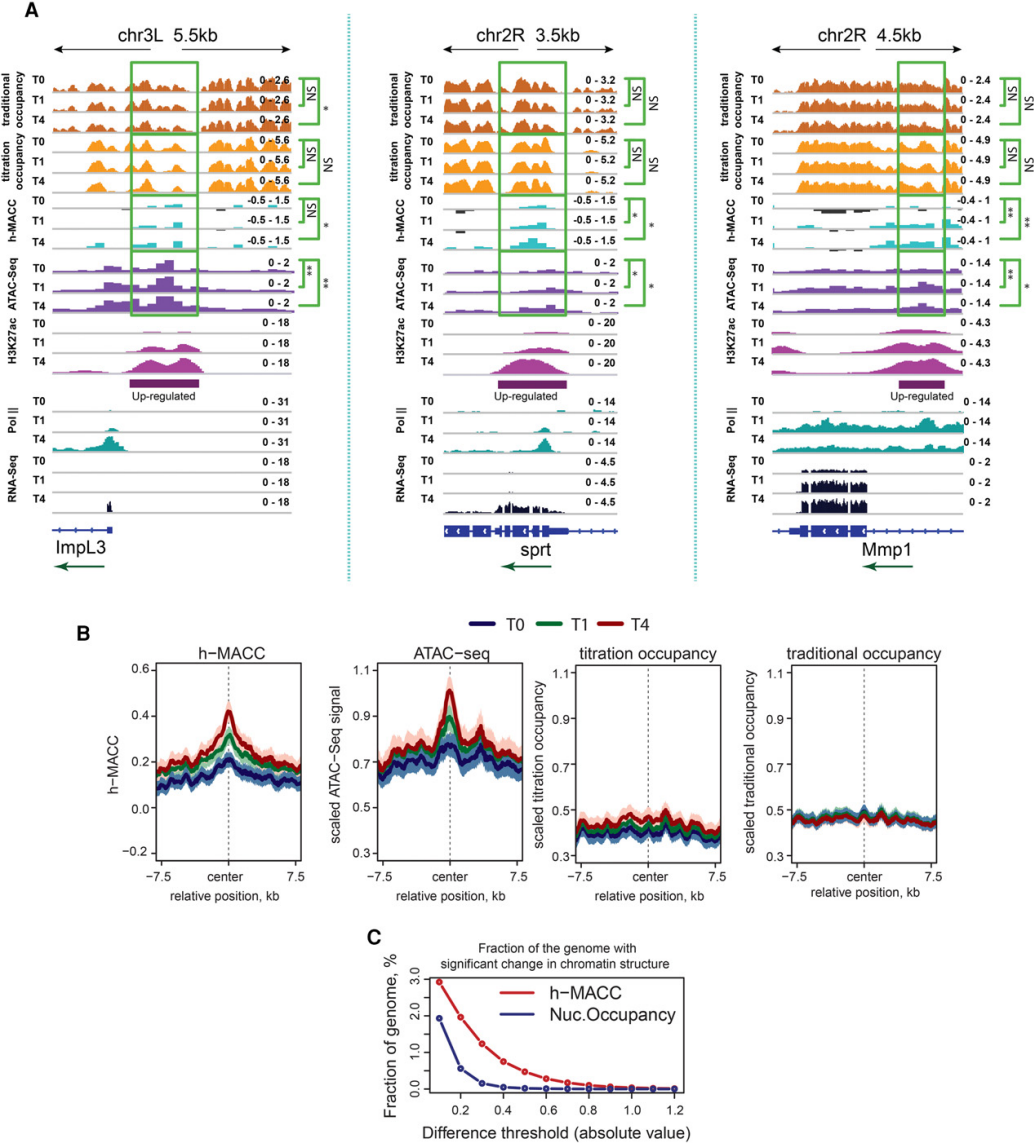
Chromatin organization at the gene-distal enhancers up-regulated in the UPR. (*A*) Profiles of nucleosome occupancy, h-MACC, ATAC-seq, H3K27ac, RNA Pol II, and RNA-seq around up-regulated enhancers. The *top* panels show nonbinned nucleosome occupancy profiles together with the h-MACC profile around the identified enhancers. *P*-values were estimated with one-sided signed rank test (see the legends for Figs. 1C and 2 for more detail). (*B*, from *left* to *right*) Average profiles of chromatin accessibility (h-MACC) and scaled profiles of ATAC-seq, titration occupancy, and traditional occupancy around up-regulated enhancers. See the legend for Figure 3A for more details. (*C*) Percentages of the genome that exhibit significant changes in either h-MACC or titration occupancy. The *Y*-axis corresponds to the percentage, and the *X*-axis corresponds to different thresholds. (Red line) Changes in h-MACC; (blue line) changes in titration occupancy.

Many of the genes up-regulated during the UPR have associated enhancer regions that show increased H3K27ac during the UPR (see the H3K27ac tracks in Fig. 2). Enhancer regions were reported to be nucleosome-depleted when active (Schaffner 2015), so we examined whether nucleosomes were lost from up-regulated enhancers. We defined up-regulated enhancers as regions that show an increase in H3K27ac levels at least twofold at time intervals 0–1 h and 1–4 h and are distinct from promoters of annotated genes (located outside the regions from −1 kb to +0.5 kb of the TSS). Examples of three of these (located in the vicinity of *ImpL3*, *sprt*, and *Mmp1*) showed that h-MACC increased over the enhancer as acetylation levels increased, and transcription increased nearby; however, nucleosome occupancy was largely unchanged (Fig. 4A). Only the *ImpL3* enhancer region additionally had regions with decreased occupancy.

We examined this more broadly across a set of 422 up-regulated enhancers. Accessibility increased from 0 h to 1 h to 4 h (Fig. 4B, left two panels). Nucleosome occupancy did not decrease as accessibility increased (Fig. 4B, right two panels). Significant changes in accessibility showed a clear trend toward the center of enhancers, whereas significant changes in occupancy were spread across the region, consistent with the hypothesis that changes in accessibility predominate as enhancers become acetylated (Supplemental Fig. S4A). We conclude that at up-regulated enhancers, the predominant change in nucleosomes was an increase in accessibility, not a decrease in occupancy.

Enhancers that showed consistently high levels of H3K27ac across the UPR time course also showed a central dip in nucleosome occupancy (Supplemental Fig. S4B). The nucleosome occupancy at these regions remained low throughout the UPR. In line with previous reports (West et al. 2014), this dip in occupancy might reflect binding of TFs prior to induction of the UPR. These observations further demonstrate that failure to capture changes in nucleosome occupancy during enhancer activation is not due to a detection issue.

To further generalize our analysis, we identified all sites where either accessibility or titration occupancy changed significantly between the 0-h and 4-h time points (*P* < 0.05, *t*-test based on the variability in replicates). The fraction of the genome that exhibited differences in accessibility was larger than the corresponding fraction estimated for nucleosome occupancy (Fig. 4C). We next tested whether our conclusions were valid even when comparisons were performed only for the peaks in occupancy profiles; i.e., for the genomic locations that are normally occupied by nucleosomes. This analysis did not require any prior binning of the genome and could detect nucleosome removal or nucleosome repositioning on a scale <200 bp. We identified all stable nucleosome positions at each time point during the UPR course (see the Materials and Methods for details). As a control, we compared changes in positions between replicates of the 0-h time point and found, as expected, that peak nucleosome positions resided close to one another in the two replicates (Supplemental Fig. S4C). If nucleosome occupancy changed during the UPR, then the values comparing 0 h with 1 h or 0 h with 4 h should show more variation in peak location than seen in the control; instead, these curves overlapped the control curve (Supplemental Fig. S4C). Thus, nucleosome positions did not “move” more between the UPR time points than the fluctuations observed between repeat experiments at the same time point. These results confirm on the genome scale our initial conclusions that nucleosome occupancy does not change significantly during acute regulation.

## Discussion

The unexpected finding in this study is that there were few changes in nucleosome occupancy across activated promoters, genes, and enhancers during a genome-wide change in regulation. Instead, we observed a significant increase in accessibility of nucleosomal DNA across these regions. Increased accessibility is normally attributed to decreased occupancy, but that correlation was not seen in our analysis. To measure accessibility and nucleosome occupancy in the same experiment, we used a recently developed assay based on MNase titration (Mieczkowski et al. 2016). This approach allowed us to determine an accessibility metric, h-MACC, that quantified the ease with which MNase released nucleosomes in any given region. This metric is capable of measuring changes in accessibility at either “open” or “closed” regions of chromatin, as it generates a value regardless of whether chromatin is accessible or inaccessible. Because this work focused on increased accessibility during activation, we were able to compare this MNase-based metric with a well-vetted measure of increased accessibility, ATAC-seq (Buenrostro et al. 2013; Buenrostro et al. 2015). While these two metrics showed good overall correlation in open regions of chromatin in the gene bodies and regulatory regions examined (Supplemental Fig. S4D), we note that MACC and ATAC-seq did not produce identical results in some gene regions (e.g., cf. the MACC and ATAC-seq panels in Fig. 1E). A systematic comparison of the two metrics would be helpful to determine the extent to which they measure similar characteristics of increased accessibility.

We observed several examples of occupancy decreases on individual up-regulated genes in gene bodies (e.g., *HSC70-3/BiP*) (Fig. 2) or promoters and enhancers (e.g., *ImpL3*) (Figs. 3B, 4A), demonstrating that the occupancy metrics detected the types of changes that have been seen previously. However, these changes were the exception rather than the rule. The lack of substantial change in occupancy was observed whether we examined individual up-regulated genes (Figs. 2, 3), up-regulated genes as a class (Fig. 1), or a sampling of the entire genome (Fig. 4).

We conclude that the primary change that occurs during this wide-scale regulation involves the characteristics of nucleosomal DNA that govern accessibility, not the location of the nucleosome. These data extend previous studies on changes in the characteristics of nucleosomes during regulation. The response of numerous genes to infection also did not show stable changes in nucleosome location but rather showed a transient change in characteristics as measured by MNase digestion (Sexton et al. 2014). Work using yeast has identified “fragile” nucleosomes at promoter regions that become highly accessible to MNase cleavage during regulation (Knight et al. 2014). The extent to which these changes are due to nucleosomes themselves as opposed to nonhistone proteins has been a matter of recent debate (Chereji et al. 2017). In larger eukaryotes, fragile nucleosomes upstream of TSSs have been found in *Drosophila* (Chereji et al. 2016, Mieczkowski et al. 2016) and human embryonic stem cells (Ishii et al. 2015; Voong et al. 2016), but whether these seemingly ubiquitous features of eukaryotic promoters are true nucleosomes or other DNA-binding proteins remains to be determined. To address whether nucleosomes were responsible for protection in the work presented here, the experiments were all performed with a histone H3 immunoprecipitation step to indicate that all measured fragments contained nucleosomes. We have no information concerning whether these fragments are also bound by other factors; in fact, binding by other factors with nucleosomes has been proposed previously to alter nucleosome accessibility. Certain TFs in mammalian cells, such as Oct4 and Sox2, were found to be targeted to nucleosomes prior to repositioning of nucleosomes, and the extent of this in vivo targeting correlated with the binding affinity of these factors to nucleosomes in vitro (Soufi et al. 2015). This extended earlier biochemistry studies on artificial templates that showed that factors differed in their ability to bind nucleosomes and help other factors gain access (Taylor et al. 1991). Only a subset of TFs has the ability to target nucleosomal DNA and was therefore termed pioneer factors (Iwafuchi-Doi and Zaret 2014). It is clear that nucleosomes are not as repressive to gene regulation as previously thought, as pioneer factors can stimulate tissue-specific gene activation; for example, FoxA is involved in creating accessibility in liver-specific enhancer regions by displacing linker histones (Iwafuchi-Doi et al. 2016).

We provide a more general analysis by examining the entire *Drosophila* genome during an acute transcriptional response and show that changes in accessibility are widespread and predominate over changes in nucleosome location. Changes in the location of nucleosomes might have been overemphasized previously due to changes in MACC that lead to perceived, rather than actual, changes in nucleosome location using single-point MNase-seq methods. We believe that this might be a general phenomenon, since we observed previously that chromatin accessibility is poorly correlated with nucleosome occupancy in unperturbed S2 cells (Mieczkowski et al. 2016). Together, these observations indicate that an increase in chromatin accessibility without a decrease in nucleosome occupancy may be a general mechanism of transcription regulation.

We present data to indicate that observed H3 occupancy was impacted by the amount of MNase used in digestions during the 4-h time course following induction of the UPR. The H3 occupancy measured with high MNase concentration at the TSSs of up-regulated genes decreases as expected for the occupancy of stable nucleosomes (Supplemental Fig. S4E). However, this decrease is compensated for by the increase of the occupancy of nucleosomes released at low MNase concentrations (accessible nucleosomes). This change in physical properties of the nucleosomes at the TSSs of the UPR-activated genes occurs without nucleosome repositioning and is reflected by the increases in the MACC or ATAC-seq signal measured in our study. We also note that the nucleosomes accessible under low MNase conditions are enriched in the “enhancer” histone mark H3K27ac (Supplemental Fig. S4F), which is consistent with increased occupancy of accessible nucleosomes at active enhancers. Thus, our results indicate that regulatory regions do not need to become nucleosome-free during a rapid transcriptional induction, suggesting that nucleosomes are not a barrier to the interactions that activate and might even be a component of the activation process involving components such as pioneer factors (Iwafuchi-Doi et al. 2016).

There are several classes of mechanisms that might lead to increased accessibility of nucleosomal DNA. Nucleosomes might become more accessible by binding of factors or loosening of the DNA via ATP-dependent remodeling. Alternatively, there might be no change in accessibility of the nucleosome itself, but rather internucleosome interactions might be weakened by altering covalent modifications or the introduction of histone variants. Finally, proteins that interact with linker DNA or both linker DNA and nucleosomes (e.g., histone H1 and HP1) might alter accessibility. We infer from our analyses that characterizing mechanisms for regulating accessibility that do not involve nucleosome movement or removal will be important for understanding how regulation occurs during the UPR and perhaps during many other types of activation.

## Materials and methods

### Experimental methods

#### UPR time course in *Drosophila* S2 cells

S2 *Drosophila* cells were grown at 28°C in Schneider's medium with 10% heat-inactivated fetal bovine serum (FBS). Cells were treated with 5 mM DTT, and 1 × 10^7^ cells were harvested at 0 h (before treatment), 1 h, and 4 h and immediately put on ice. Cells were then cross-linked and stored as described previously (Mieczkowski et al. 2016).

#### MNase titration

MNase titration was performed as described previously (Mieczkowski et al. 2016).

For h-MACC, after addition of EDTA/EGTA and SDS, half of the digests were kept at 4°C as input, and the other half were adjusted to chromatin immunoprecipitation (ChIP) buffer conditions (10 mM TRIS at pH 8.0, 100 mM NaCl, 1 mM EDTA, 0.1% sodiumdeoxycholate, 0.5% sarkosyl, 1% Triton X-100, complete protease inhibitors [Roche]) with 1 mL of ChIP buffer. After tumbling for 10 min at 4°C, the digests were spun at high speed for 10 min at 4°C, and the supernatant was incubated with anti-H3 antibodies (Abcam, ab1791) as described (Bowman et al. 2013; Mieczkowski et al. 2016).

DNA was analyzed on the BioAnalyzer and then used directly for library preparation as described previously (Bowman et al. 2013; Mieczkowski et al. 2016). Libraries were sequenced with Paired-End 50 on a HiSeq 2000 (Illumina).

#### RNA-seq

RNA preparation from 10^7^ cells was performed as described previously (Mieczkowski et al. 2016).

#### ChIP for H3K27ac and RNA Pol II

From each treatment group, 3 × 10^7^ to 6 × 10^7^ cross-linked cells were resuspended in sonication buffer (0.5% SDS, 20 mM Tris at pH 8.0, 0.5 mM EGTA, 2 mM EDTA, protease inhibitor tablets [Roche]) in a ratio of 100 µL of cold sonication buffer to 1 × 10^7^ cells. Cells were lysed for 10 min on ice. Cell lysates were sonicated in a Qsonica at 4°C. After a hard spin for 10 min at 4°C, the supernatants corresponding to 2.5 million cells were removed and either flash-frozen and kept at −80°C (as input) or diluted into 1 mL of ChIP buffer (0.5% Triton X-100, 2 mM EDTA, 20 mM TRIS at pH 8.0, 150 mM NaCl, 10% glycerol) to be used for ChIP after a hard spin for 10 min at 4°C. The immunoprecipitation solution was incubated with the respective immunoprecipitation antibody overnight at 4°C with tumbling. The next day, protein A Dynabeads (Life Technologies) were added, and, after a 2-h incubation while tumbling at 4°C, the immunoprecipitation samples were washed once with low-salt wash buffer (0.1% SDS, 1% Triton X-100, 2 mM EDTA, 20 mM Tris at pH 8, 150 mM NaCl), three times with high-salt wash buffer (same as the previous buffer but with 500 mM NaCl), and once with LiCl wash buffer (0.25 M LiCl, 1% NP-40, 1% NADeoxycholate, 1 mM EDTA, 10 mM Tris at pH 8). The beads were rinsed with TE buffer, and the proteins were eluted with 500 µL of elution buffer (1% SDS, 0.1 M NaHCO_3_) for 30 min at room temperature. At this point, the input samples were also taken up in elution buffer.

Downstream experiments were performed as described previously (Mieczkowski et al. 2016).

#### Antibodies

The antibodies used were anti-Pol II (Abcam, ab5131), anti-H3K27ac (Active Motif, 39136), and anti-H3 (Abcam, ab1791).

#### ATAC-seq

The UPR time course was performed on S2 cells as described above. From each time point, 5 × 10^4^ cells were collected and washed in cold PBS. ATAC-seq libraries were prepared as described previously (Buenrostro et al. 2013, 2015). Briefly, cells were resuspended in cold lysis buffer and centrifuged to collect nuclei. Transposition reaction was performed for 30 min at 37°C using Tn5 transposase from Nextera. DNA fragments were purified using a Minelute kit (Qiagen). DNA fragments were amplified using Nextera barcoded primers as described. The amplified library was purified with SPRI beads. Paired-End 50 sequencing was done on an Illumina HiSeq2000 according to the manufacturer's instructions.

### Data processing and statistical analysis

#### Sequencing data alignment

Two replicates of each data set containing between ∼20 million and 40 million paired-end fragments were produced (Supplemental Table S1). The sequenced paired-end reads were mapped to the *Drosophila melanogaster* genome (dm3) using Bowtie aligner version 0.12.9 (Langmead et al. 2009) for the MNase-seq, ChIP-seq (ChIP combined with high-throughput sequencing), and ATAC-seq data. Only uniquely mapped reads were retained. The reads with the insert sizes <50 bp or >500 bp were removed from further analysis. Genomic positions with the numbers of mapped tags above the significance threshold of *Z*-score of 7 were identified as anomalous, and the tags mapped to such positions were discarded (Tolstorukov et al. 2009). RNA-seq reads were aligned using the TopHat software package for dm3 genome assembly with default parameters (Trapnell et al. 2009).

#### Computation of MACC scores

MACC profiles were computed as described previously (Mieczkowski et al. 2016). Briefly, read frequencies were computed in nonoverlapping bins of a selected size (200 bp for most analyses) for each titration point independently and normalized to library sizes. A linear regression was fitted on the normalized frequencies in bins. The estimated regression coefficients were corrected to remove dependence on the GC content of underlying sequences, and these values were used as MACC scores.

#### Processing MNase-seq and ATAC-seq data

H3 occupancy was estimated as tag frequency averaged over all four titration points either in bins of the same size as the bins used for MACC estimation (200 bp) for direct comparison of MACC and H3 occupancy (e.g., Fig. 1E) or without bins for other analyses. To validate our data normalization procedure, we checked that the H3 occupancy did not change during the UPR in the genes that did not show considerable changes in expression (Supplemental Fig. S1F). We also compared the H3 tag frequency profiles averaged over a set of ∼7000 randomly selected sites, which corresponded to the number of the expressed genes identified in this study (Supplemental Fig. S1D, left panels), and confirmed that H3 levels were similar for all titration and time points as expected (Supplemental Fig. S4G). Input correction was not used in most analyses in this study; however, similar results were obtained when either H3 enrichment (ChIP/input) or input-subtracted H3 frequency was used (cf. Fig. 1E and Supplemental Figs. S1E,F). Traditional nucleosome occupancy was computed in a similar way using the data generated for the samples where different digests were pooled before sequencing. For consistency, ATAC-seq signal was summarized in the same bins as MNase-seq data. The ATAC-seq, H3 occupancy, and traditional nucleosome occupancy values in bins were further scaled to facilitate the comparison with h-MACC. Specifically, the values of each metric were scaled so that median values of the first and 20th quantiles were scaled between 0 and 1. To confirm that our findings did not depend on the scaling or binning procedures, the main results were reproduced with the unscaled metrics. In particular, tag coverage was computed for nucleosome and H3 occupancy data as well as ATAC-seq data as the library size-normalized number of paired-end fragments spanning over each genomic location.

#### Identification of differentially expressed genes

RNA-seq tag frequencies were normalized for GC content using Bioconductor package EDASeq, and the expression estimates for each gene were obtained using Bioconductor package DESeq (Risso et al. 2011; Anders et al. 2013). Differentially expressed genes were called using limma package (Ritchie et al. 2015). Genes that had significant (Benjamini and Hochberg-corrected *P* < 0.05) changes in their expression levels more than twofold at the time intervals 0–1 h and 0–4 h were called as differentially expressed.

#### Identification of putative enhancers

The Spp package (Kharchenko et al. 2008) was used to identify regions enriched in H3K27ac over input. For each of the analyzed samples, a 200-bp window and *Z*-threshold of 10 were used to select the H3K27ac-enriched regions. The selected enriched regions closer to each other than 150 bp were joined. Only regions enriched in both replicates within the same time point were preserved and identified as enhancers. Enhancers located closer than 1 kb upstream of TSSs or 500 bp downstream from TSSs were excluded from the analysis of TSS-distal enhancers. The enhancers were classified as “up-regulated” if the corresponding H3K27ac enrichment significantly increased twofold over the time intervals 0–1 h and 0–4 h. The enhancers that did not exhibit significant twofold changes in the H3K27ac levels but had the H3K27ac enrichment (ChIP/input) above fourfold at all time points were identified as those having higher levels of H3K27ac at all time points.

All sequencing data are available under Gene Expression Omnibus accession numbers number GSE95689.

## Supplementary Material

Supplemental Material
