# Supplementary material for: Widespread changes in nucleosome accessibility without changes in nucleosome occupancy during a rapid transcriptional induction
Source: Genes Dev. 2017 Mar 1;31(5):451–62. doi: 10.1101/gad.293118.116 (PMC5393060; doi:10.1101/gad.293118.116)
Supplement: Supplemental Material [file supp_31_5_451__index.html]

Widespread changes in nucleosome accessibility without changes in nucleosome occupancy during a rapid transcriptional induction — Supplemental Material 

# Widespread changes in nucleosome accessibility without changes in nucleosome occupancy during a rapid transcriptional induction

## Supplemental Material

- Supplemental\_Data.pdf
