# Supplementary material for: Widespread changes in nucleosome accessibility without changes in nucleosome occupancy during a rapid transcriptional induction
Source: Genes Dev. 2017 Mar 1;31(5):451–62. doi: 10.1101/gad.293118.116 (PMC5393060; doi:10.1101/gad.293118.116)
Supplement: Supplemental Material [file supp_gad.293118.116_Supplemental_Data.pdf]

Supplementary figures

Figure S1

(A) Schematic illustration of UPR induction. Three sensors for misfolded proteins have been identified: the IRE1α, ATF6, and PERK. IRE1α is a transmembrane ER serine/threonine kinase maintained in monomeric form by association with the ER chaperone BiP (Cox et al. 1993; Bertolotti et al. 2000). When unfolded proteins accumulate in the ER, BiP dissociates from IRE1α. This allows IRE1α to homodimerize and autophosphorylate (Shamu and Walter 1996), an event that activates the cytosolic endoribonuclease activity of IRE1α. This activity removes an intron from the mRNA that encodes the transcription factor XBP-1 (X-BOX binding protein 1 (Kawahara et al. 1998)), followed by a tRNA-ligase-mediated reaction to yield the functional XBP-1 mRNA. ATF6 is cleaved upon titration of BiP by excess unfolded proteins (Ye et al. 2000; Shen et al. 2002), and generates the transcriptional activator ATF6f, which upregulates ER chaperones and XBP-1 (Yoshida et al. 2001; Lee et al. 2003). PERK activity decreases overall translation to decrease protein overload (Harding et al. 2000), and induces the translation of ATF4, another UPR-specific transcription factor that can heterodimerize with XBP-1.The transcription factors (XBP-1, ATF6f, and ATF4) involved in the UPR are bZIP proteins that might hetero- and homo-dimerize through their leucine zipper domains (Bernales et al. 2006). They bind to either the ER stress response element (ERSE I-III) or to the UPR response element (UPRE1-3).
(B) RT-PCR agarose gel used to select time points. S2 cells were treated with 5mM DTT, 300nM Thapsigargin (TG), or 10ug/ml tunicamycin (TM). Cells were harvested at the indicated times, RNA was prepped and RT-PCR was performed with primers specific for drosophila XBP-1 (Plongthongkum et al. 2007). Bands were resolved on a 1.5% agarose gel and visualized with Etidium Bromide.
(C) Gene expression levels in the sets of up- and down-regulated genes. Left panel: Heatmap representing expression of down- and up-regulated genes over three time points. Expression values computed for independent replicates were used for this

analysis . We note that the analyzed samples have grouped according to time points in unsupervised clustering (see dendrogram at the top). Blue, green and red lines below the heatmap correspond to the time points 0h, 1h, and 4h respectively. Right panel: Distributions of average expression levels within time points. Blue, green and red boxplots correspond to the time points 0h, 1h, and 4h respectively.

(D) MNase-seq profiles around TSS (transcription start sites) for expressed (left) and silent (right) genes. Upper, middle and bottom panels show profiles corresponding to time points 0h, 1h and 4h respectively. Yellow-blue colour scheme indicates MNase concentration levels (1.5, 6.25, 25 and 100 U), with bright yellow corresponding to the lowest concentration and dark blue corresponding to the highest. The red line depicts an averaged profile.

(E) Per-gene levels of the input corrected titration occupancy measured over gene bodies of up-regulated genes. The plot is organized as a panel of Figure 1E.

(F) Per-gene levels of the MNase chromatin accessibility, ATAC-seq, titration occupancy, 'traditional' occupancy and input corrected titration occupancy over gene bodies computed for the genes that do not much change expression (<20%) between time points. Blue, green and red boxplots show results for time points 0h, 1h and 4h respectively. Significance of the changes was evaluated with Mann-Whitney test.

(G) Profiles of nucleosome occupancy, h-MACC, ATAC-seq, H3K27ac enrichment, RNA Pol II and RNA-seq at gene bodies of selected genes from Hsp region. See legend of Figure 2 for more details. Directions of transcription are marked with green arrows.

Figure S2

(A) The 200-bp binned profiles of nucleosome occupancy corresponding to Figure 2. To facilitate the metric-to-metric comparison the profiles were shown at the same genomic range as the accessibility metrics on Figure 2. Green boxes show the loci for which the statistical test was performed.

(B) Distribution of gene expression log-fold changes between T4 and T0 stratified according to titration occupancy (left) or h-MACC (right) changes. The red dots

correspond to medians in each subset. P-values shown above the plots were estimated with the Mann-Kendall trend test.

Figure S3

(A) Average profiles of un-scaled ATAC-seq and occupancy profiles around TSS of up-regulated genes. For details please see legend of Figure 3A.

(B) Correlations of gene expression levels with h-MACC and titration occupancy values around TSS of up-regulated genes. Correlations values were computed between gene expression estimations (RNA-seq data) and corresponding the values of h-MACC or titration occupancy averaged over [TSS-1kb,TSS+1kb] regions. Purple and orange bars show correlation coefficient with h-MACC and titration occupancy in the analyzed time points respectively.

(C) From left to right: Average profiles of chromatin accessibility (h-MACC), and scaled profiles of ATAC-Seq, titration occupancy, and 'traditional' occupancy over down-regulated genes. See legend to Figure 3A for more details.

(D) Profiles of nucleosome occupancy, h-MACC, ATAC-seq, H3K27ac, RNA Pol II and RNA-seq at the selected up-regulated genes. Transcription direction is marked with green arrows. The entire gene body is shown in each individual example. As previously (see legend to Figure 2 for details) ATAC-seq was scaled so that its dynamic ranges became similar to that of h-MACC.

Figure S4

(A) Heatmaps showing change of metrics around up-regulated enhancers in the time interval 0h-4h. The metrics appear in the same order as in Figure 4B. Red and blue colors correspond to increase and decrease in a metric respectively. Each row represents individual enhancer. Rows in all heatmaps were ordered by the magnitude of the h-MACC change at the enhancers.

(B) Titration occupancy and 'traditional' occupancy profiles around positions with the strongest H3K27ac signals ('peaks') within enhancers exhibiting high levels of H3K27ac enrichment at all UPR time points. Blue, green and red colors correspond to time points 0h, 1h, and 4h time points.

(C) Similarity of stable nucleosome positions in the samples corresponding to different UPR time points. The six plots correspond to different experimental setup (titration and 'traditional' nucleosome occupancies profiles appear in the upper and lower rows respectively) and different genomic locations (form left to right: TSS of up-regulated genes, up-regulated enhancers and entire genome). We identified all stable nucleosome positions in all replicates at each time point during the UPR course using un-binned data (see Methods for details), and compared the detected positions with those identified for the replicate 1 at time point 0h. The plots show the fractions of nucleosome positions (Y-axis) within a given distance (X-axis) from the positions in the replicate 1 at time point 0h. Randomly selected positions in the analyzed regions were used for reference. Blue color represents the second replicate at time point 0h; green and red colors represents both replicates at time points 1h and 4h respectively; grey color represents randomized nucleosome positions. We note that the curves obtained for the samples corresponding to the different time points are similar to those corresponding to comparison of the replicates obtained at the same time point 0h.

(D) Correlations between h-MACC and ATAC-seq in the accessible regions (positive values of h-MACC). X-axis corresponds to h-MACC and Y-axis corresponds to ATAC-seq. The three panels from left to right show correlations computed for T0, T1 and T4 time points respectively. Pearson's and Spearman's correlation coefficients are shown above the plots.

(E) Profiles representing differences between MNase fragment frequencies around TSS of up-regulated genes measured in 0h or 4h time points. Yellow and blue lines correspond to the 1.5 U and 100 U MNase concentrations respectively.

(F) MNase fragment frequencies obtained with low (1.5 U, yellow) and high (100 U, blue) MNase concentrations with regard to H3K27ac enrichment levels. 10% of the genome with the lowest and 10% with the highest H3K27ac enrichments were used in this analysis. The left and the right panels correspond to profiles measured at 0h and 4h time points respectively.

(G) MNase-seq profiles around randomly selected locations. See legend of Supplementary Figure 1D for more details.

References

Bernales S, Papa FR, Walter P. 2006. Intracellular signaling by the unfolded protein response. *Annual review of cell and developmental biology* **22**: 487-508.

Bertolotti A, Zhang Y, Hendershot LM, Harding HP, Ron D. 2000. Dynamic interaction of BiP and ER stress transducers in the unfolded-protein response. *Nature cell biology* **2**: 326-332.

Cox JS, Shamu CE, Walter P. 1993. Transcriptional induction of genes encoding endoplasmic reticulum resident proteins requires a transmembrane protein kinase. *Cell* **73**: 1197-1206.

Harding HP, Novoa I, Zhang Y, Zeng H, Wek R, Schapira M, Ron D. 2000. Regulated translation initiation controls stress-induced gene expression in mammalian cells. *Molecular cell* **6**: 1099-1108.

Kawahara T, Yanagi H, Yura T, Mori K. 1998. Unconventional splicing of HAC1/ERN4 mRNA required for the unfolded protein response. Sequence-specific and non-sequential cleavage of the splice sites. *The Journal of biological chemistry* **273**: 1802-1807.

Lee AH, Iwakoshi NN, Glimcher LH. 2003. XBP-1 regulates a subset of endoplasmic reticulum resident chaperone genes in the unfolded protein response. *Molecular and cellular biology* **23**: 7448-7459.

Plongthongkum N, Kullawong N, Panyim S, Tirasophon W. 2007. Ire1 regulated XBP1 mRNA splicing is essential for the unfolded protein response (UPR) in Drosophila melanogaster. *Biochemical and biophysical research communications* **354**: 789-794.

Shamu CE, Walter P. 1996. Oligomerization and phosphorylation of the Ire1p kinase during intracellular signaling from the endoplasmic reticulum to the nucleus. *The EMBO journal* **15**: 3028-3039.

Shen J, Chen X, Hendershot L, Prywes R. 2002. ER stress regulation of ATF6 localization by dissociation of BiP/GRP78 binding and unmasking of Golgi localization signals. *Developmental cell* **3**: 99-111.

Ye J, Rawson RB, Komuro R, Chen X, Dave UP, Prywes R, Brown MS, Goldstein JL. 2000. ER stress induces cleavage of membrane-bound ATF6 by the same proteases that process SREBPs. *Molecular cell* **6**: 1355-1364.

Yoshida H, Matsui T, Yamamoto A, Okada T, Mori K. 2001. XBP1 mRNA is induced by ATF6 and spliced by IRE1 in response to ER stress to produce a highly active transcription factor. *Cell* **107**: 881-891.

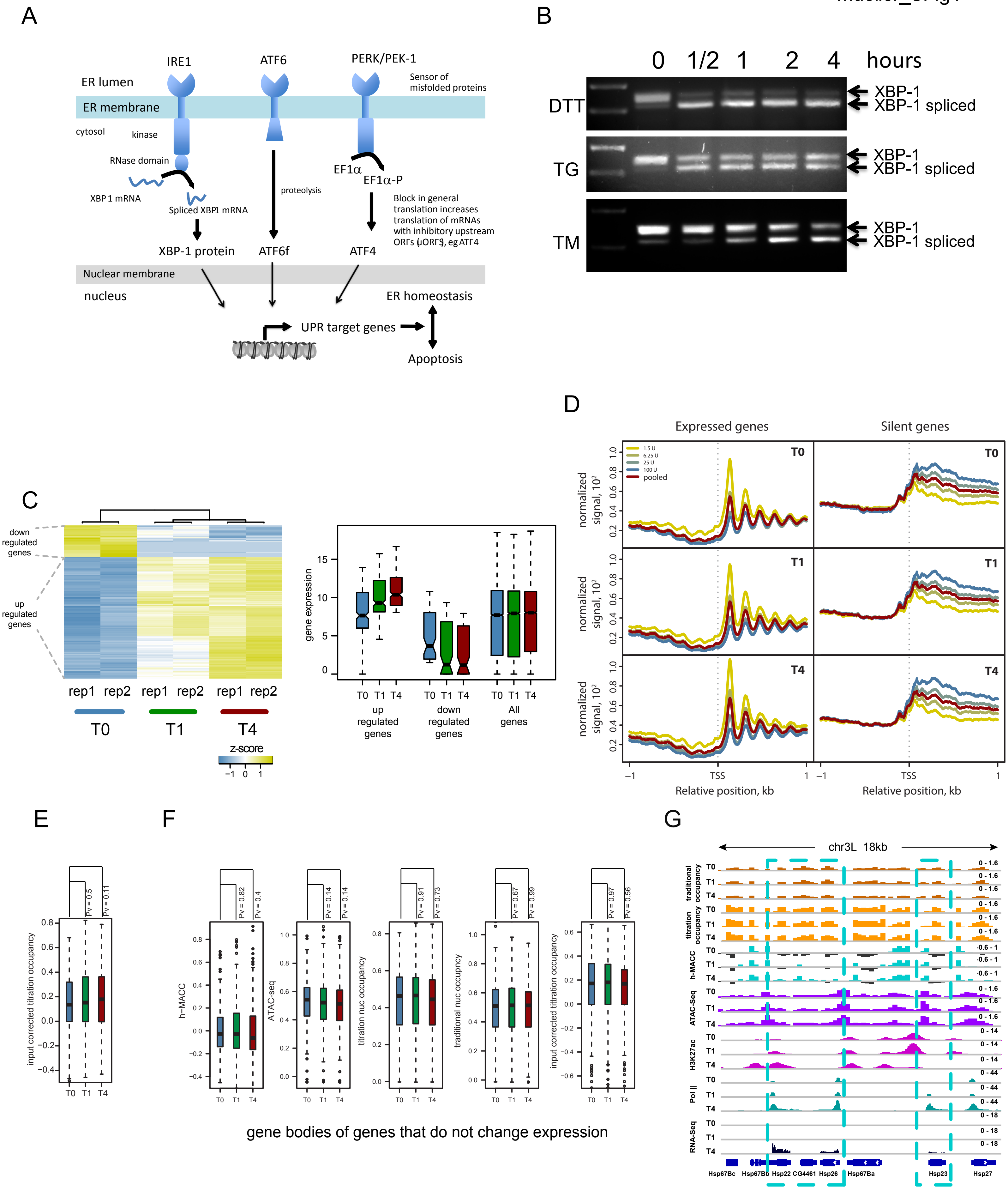
A
IRE1
ATF6
PERK/PEK-1
ER lumen
Sensor of misfolded proteins
ER membrane
cytosol
kinase
RNase domain
XBP-1 mRNA
Spliced XBP1 mRNA
proteolysis
EF1α
EF1α-P
Block in general translation increases translation of mRNAs with inhibitory upstream ORFs (uORFs), eg ATF4
XBP-1 protein
ATF6f
ATF4
Nuclear membrane
nucleus
ER homeostasis
UPR target genes
Apoptosis
B
0 1/2 1 2 4 hours
DTT
TG
TM
XBP-1
XBP-1 spliced
C
down regulated genes
up regulated genes
rep1 rep2 rep1 rep2 rep1 rep2
T0 T1 T4
z-score
gene expression
up regulated genes
down regulated genes
All genes
D
Expressed genes
Silent genes
1.5 U
6.25 U
25 U
100 U
pooled
normalized signal, 10²
TSS
Relative position, kb
E
input corrected titration occupancy
F
h-MACC
ATAC-seq
titration nuc occupancy
traditional nuc occupncy
input corrected titration occupancy
gene bodies of genes that do not change expression
G
chr3L 18kb
traditional occupancy
titration occupancy
h-MACC
ATAC-Seq
H3K27ac
Pol II
RNA-Seq
Hsp67Bc
Hsp67Bb
Hsp22
CG4461
Hsp26
Hsp67Ba
Hsp23
Hsp27

Mueller_SFig2

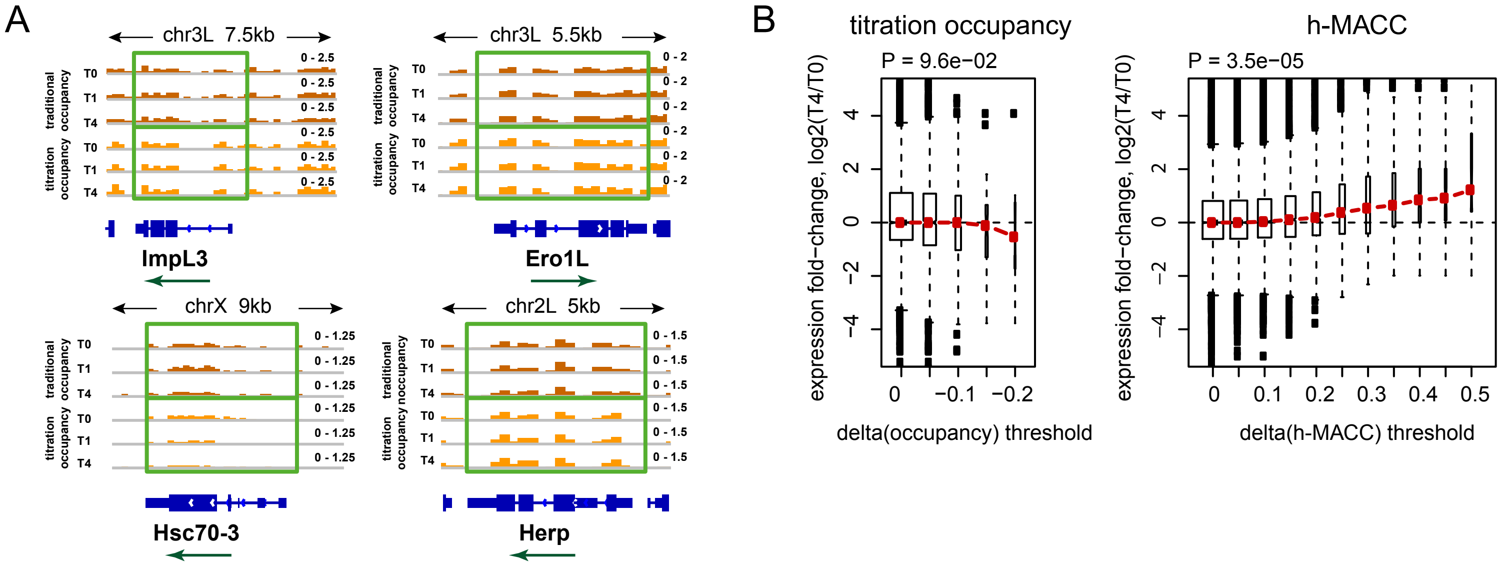

Mueller_SFig3

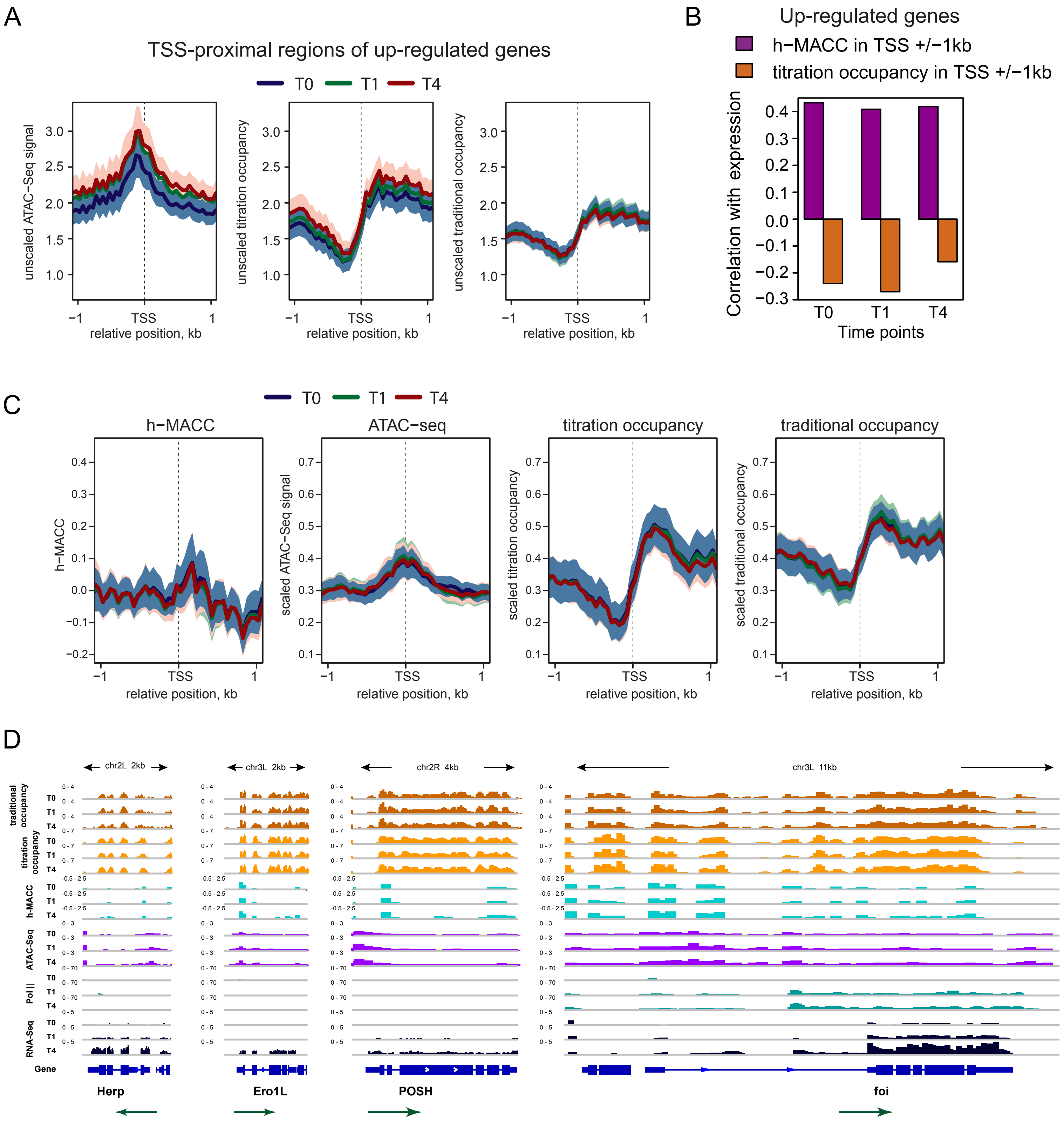

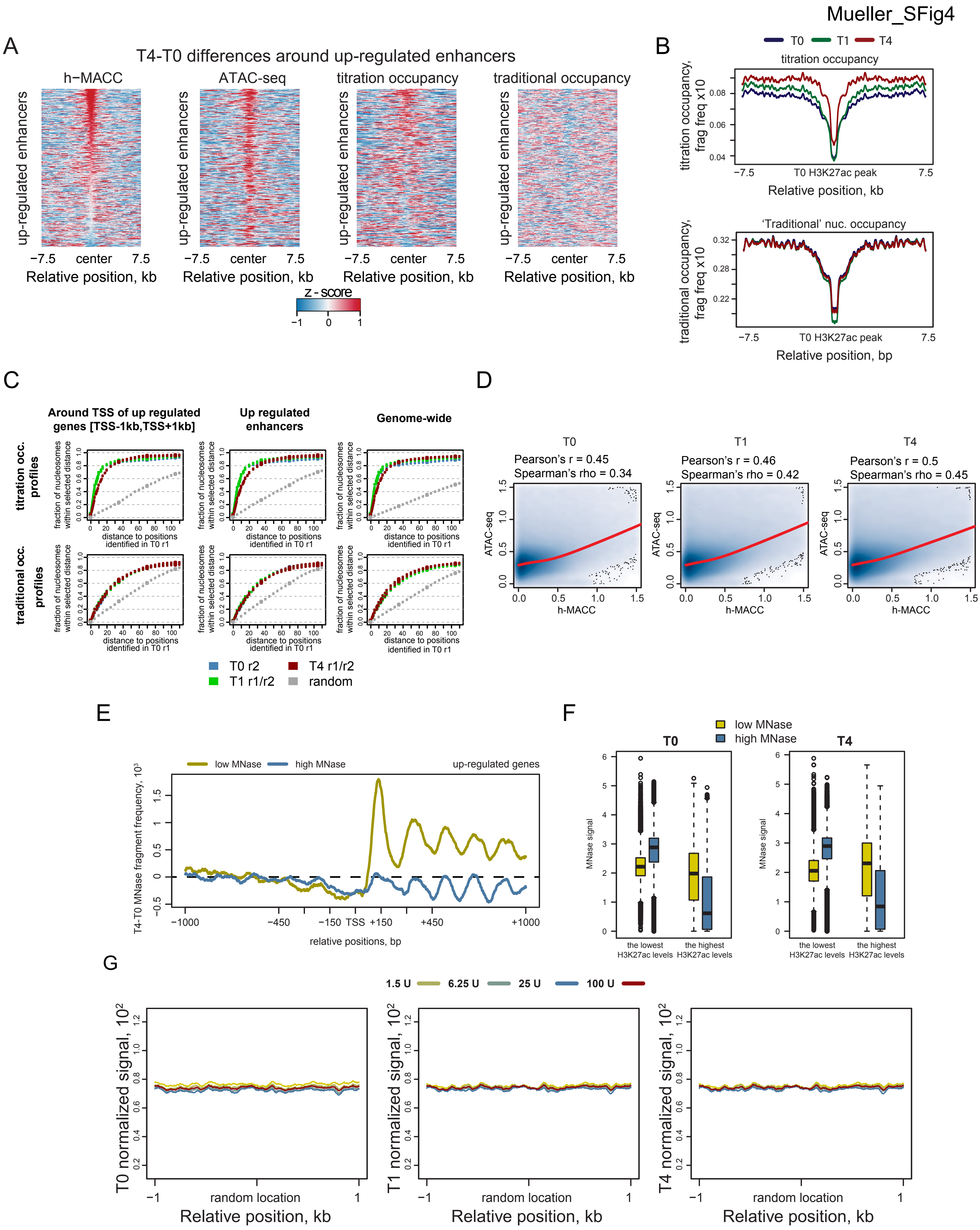
Mueller_SFig4
A
T4-T0 differences around up-regulated enhancers
h−MACC
ATAC-seq
titration occupancy
traditional occupancy
up-regulated enhancers
−7.5 center 7.5
Relative position, kb
z - score
−1 0 1
B
T0 T1 T4
titration occupancy
titration occupancy, frag freq x10
0.08
0.06
0.04
−7.5 T0 H3K27ac peak 7.5
Relative position, kb
'Traditional' nuc. occupancy
traditional occupancy, frag freq x10
0.32
0.28
0.22
Relative position, bp
C
Around TSS of up regulated genes [TSS-1kb,TSS+1kb]
Up regulated enhancers
Genome-wide
titration occ. profiles
traditional occ. profiles
fraction of nucleosomes within selected distance
distance to positions identified in T0 r1
T0 r2
T1 r1/r2
T4 r1/r2
random
D
T0
Pearson's r = 0.45
Spearman's rho = 0.34
T1
Pearson's r = 0.46
Spearman's rho = 0.42
T4
Pearson's r = 0.5
Spearman's rho = 0.45
ATAC-seq
h-MACC
E
low MNase
high MNase
up-regulated genes
T4-T0 MNase fragment frequency, 10³
−1000 −450 −150 TSS +150 +450 +1000
relative positions, bp
F
low MNase
high MNase
T0
T4
MNase signal
the lowest H3K27ac levels
the highest H3K27ac levels
G
1.5 U
6.25 U
25 U
100 U
T0 normalized signal, 10²
T1 normalized signal, 10²
T4 normalized signal, 10²
−1 random location 1
Relative position, kb

Supplementary Table 1. Counts of aligned sequenced reads in the analyzed data sets.

titration occupancy

	T0	T1	T4
H3 ChIP 1.5 U replicate 1	27,307,111	36,458,830	28,192,548
H3 ChIP 6.25 U replicate 1	32,957,126	39,200,649	31,759,201
H3 ChIP 25 U replicate 1	39,765,356	42,310,498	34,710,355
H3 ChIP 100 U replicate 1	29,507,362	38,348,773	30,904,276
H3 ChIP 1.5 U replicate 2	28,998,269	35,180,428	33,360,463
H3 ChIP 6.25 U replicate 2	34,855,344	25,927,891	31,263,089
H3 ChIP 25 U replicate 2	33,563,883	40,743,748	29,206,130
H3 ChIP 100 U replicate 2	33,698,635	34,398,993	27,527,252

ATAC-seq

	T0	T1	T4
replicate 1	34,720,622	36,944,469	35,902,454
replicate 2	27,212,528	26,731,263	25,445,928

H3K27ac

	T0	T1	T4
ChIP replicate 1	15,975,409	16,066,765	19,913,070
ChIP replicate 2	23,213,683	24,263,938	19,463,291
input replicate 1	25,692,560	24,586,658	16,163,566
input replicate 2	23,658,519	24,058,303	24,010,492

RNA-seq

	T0	T1	T4
replicate 1	19,058,611	17,203,709	17,284,586
replicate 2	16,496,790	16,393,189	15,094,682
